# Editorial: Fungal Wheat Diseases: Etiology, Breeding, and Integrated Management

**DOI:** 10.3389/fpls.2021.671060

**Published:** 2021-03-30

**Authors:** María Rosa Simón, Andreas Börner, Paul C. Struik

**Affiliations:** ^1^Cerealicultura, Facultad de Ciencias Agrarias y Forestales, Universidad Nacional de La Plata, La Plata, Argentina; ^2^Consejo Nacional de Investigaciones Científicas y Técnicas La Plata, La Plata, Argentina; ^3^Genebank Department, Leibniz Institute of Plant Genetics and Crop Plant Research, Seeland, OT Gatersleben, Germany; ^4^Department of Plant Sciences, Centre for Crop Systems Analysis, Wageningen University & Research, Wageningen, Netherlands

**Keywords:** breeding for resistance, durability of resistance, pathogen populations, resistance location, integrated management, yield, quality, wheat

## Introduction to Wheat Diseases

Agriculture in 2050 will need to produce about 50% more food because of the increase in the world population and the change in diets (FAO, 2017). Wheat production should increase, as it is one of the main staple crops in the world, providing 20% of calories and proteins for human nutrition (Tilman et al., 2011); this growth will be mainly based on yield increases, as there is strong competition for scarce productive arable land from other sectors in society (FAOSTAT, 2020). Future demand will need to be achieved through sustainable growth combining integrated management of diseases and pests, adaptation to warmer climates and increased frequency of abiotic stresses, and reduced use of water and other resources. Among the biotic constraints, Savary et al. (2019) estimated that 21.5% of current yield losses are due to pests and diseases. Of the 31 pest and pathogens reported in wheat, fungal diseases as leaf rust, Fusarium head blight, Septoria leaf blotch, stripe rust, spot blotch, tan spot, and powdery mildew cause the most serious losses. Wheat diseases also cause alterations in chemical properties and quality (Gaju et al., 2014).

This special issue consists of 26 papers, including six reviews, one mini-review, 18 original papers and one methodology paper. Recent findings on the effects of fungal wheat diseases on yield and quality, modern methods for molecular diagnosis, the variability of pathogen populations and mechanisms for infecting the wheat crop were addressed. The state of the art in breeding for resistance, new tools to locate genes and quantitative trait loci (QTL), methods for cloning resistant genes and avirulent effectors, biological and chemical control, and the release of improved cultivars were also addressed.

## Effects of Wheat Diseases on Yield Generation and Quality

In this special issue, Simón et al. reviewed the impact of nitrogen fertilization and fungicides on the severity of foliar diseases and their effects on biomass accumulation, grain yield, milling, and end-use quality, as affected by the bread-making characteristics of the wheat genotypes and the nutritional requirements of the pathogen involved. Differential effects of three main pathogens representative of biotrophic, hemibiotrophic, and necrotrophic nutritional habits on nitrogen dynamics and quality of wheat and some general models of their effects are outlined.

Surovy et al. found that *Magnaporthe oryzae* B. C. Couch (anamorph *Pyricularia oryzae* Cavara), *Triticum* pathotype affected grain characteristics and seed germination. The protein content strongly increased, whereas grain moisture, lipid and ash concentrations slightly increased as a result of disease infection. Total antioxidant activity, flavonoid, and carotenoid concentrations significantly decreased under increasing blast severity. Moreover, grain K and total phenolic concentration were augmented at a certain level of severity and then reduced, whereas some other biochemical traits increased or decreased.

*Fusarium graminearum* (Schwabe), the causal agent of Fusarium head blight, is a problem for millers as it contaminates wheat grains with mycotoxins, such as deoxynivalenol (DON) (Andersen, 1948). One of the major problems in controlling the disease is the poor efficacy due to the lack of uniformity among the different plants in the field at the beginning of anthesis when fungicides must be applied. Rod et al. found that in-furrow phosphorus application had no effect on anthesis uniformity or DON levels. Harvesting at 20–22% moisture reduced Fusarium damaged kernel ratings and percent kernel infection but increased DON levels compared with harvesting at 13–15% moisture. The highest seeding rate used advanced the date of anthesis in a late-planted crop, and decreased Fusarium head blight incidence, but did not reduce DON contamination.

## Etiology and Variability of Pathogen Populations

In the context of integrated disease management, the precise characterization of each pathogen using molecular techniques is crucial. In this special issue, Kashyap et al. developed a simple, fast and very specific molecular assay for the detection of *Urocystis agropyri* (G. Preuss) J. Schröter, the causal agent of flag smut, which showed 100% reliability.

Furthermore, the study of the variability and changes in pathogen populations is a pre-requisite for releasing cultivars with gene combinations that adequately control the predominant pathotypes. In this sense, Figlan et al. reviewed the variability and distribution of the *Puccinia triticina* Eriks. population, the causal agent of leaf rust and the scenarios of breeding wheat cultivars with durable resistance in southern Africa. Moreover, the applications of molecular markers and field pathogenomics tools using new DNA technologies are presented. How the recent availability of a high-quality reference genome of this pathogen and new DNA/RNA technologies will help to profile the gene expression of both the pathogen and the wheat crop is also described.

Skolotneva et al. studied the population composition of *Puccinia graminis* f.sp. *tritici* Eriks. and E. Henn, the causal agent of stem rust, in Siberia, where the genetic basis of resistance to the pathogen is limited. This paper found that most of the Siberian isolates collected were virulent to wheat lines with genes *Sr5, Sr9a, Sr10, Sr38, SrMcN*, and avirulent to lines with genes *Sr24, Sr3*. They also determined the ability of several genes to distinguish between the regional populations.

## Integrated Management of Wheat Diseases

Prediction of epidemics is a relevant tool to develop proper integrated management strategies when the risk is high before the season starts. Among those strategies, acceptable levels of resistance and its mechanisms, cultural practices, such as adequate fertilization management the use of crop rotation, time of planting as well as tillage increasing residue decomposition (Simón et al., 2011) are important in reducing diseases intensity. Biological and chemical control are also key tools to reduce the disease severity minimizing yield losses. In this special issue, Kim and Choi performed a retrospective study based on an epidemic of wheat blast in Bangladesh and calibrated a model to determine the viability of seasonal wheat blast risk predictions for interventions to reduce epidemics.

## Mechanisms of Resistance to Wheat Diseases

The study of the mechanisms of resistance to diseases is essential to fully understand the interactions between pathogens and the crop. In this special issue, Mohammadi et al. showed the significant biological function of the *ZtR1m1* gene, encoding a MADS-box transcription factor in the last stage of infection of *Zymoseptoria tritici* P. Crous, the causal agent of Septoria leaf blotch, indicating that *ZtR1m1* affects penetration, colonization, fungal biomass production, pycnidial formation, differentiation, and melanization required for fruiting body formation.

Furthermore, black point is a disease generating dark discoloration of the embryo of kernels, causing a reduction in grain yield, seed germination and seedling growth (Conner et al., 1996). Discoloration can be due to fungal colonization of the causal agents, such as *Bipolaris sorokiniana* (Sacc.) Shoemaker [teleomorph *Cochliobolus sativus* (Ito and Kuribayashi) Drechs. ex Dastur], *Alternaria alternata* (Fries) Keissler and Fusarium spp. (Kumar et al., 2002) or to biochemical changes produced by the pathogens and environmental stress (Walker, 2011). In this special issue, Li et al. using metabolomics and spectroscopic analysis found that blackening was produced by enzymatic browning instead of by the causal fungus.

Nilsen et al. found cell wall thickening within the rachis node and visible hyphae in the spikelets inoculated with *Fusarium graminerarum*, but restricted to the inoculation point in a resistant cultivar. Furthermore, the rachis node below the inoculation point in the resistant cultivar showed fewer differentially expressed genes compared with the non-inoculated control. Differentially expressed genes in the resistant and the susceptible cultivars resulted in the discovery of the activation of genes in response to infection. High nucleotide diversity in chromosomes, such as on 5AL differentiating both cultivars and also genes on that chromosome affecting pathways involved in the infection process and the production of DON by the fungus were identified. Fabre et al. reported that some crop mechanisms and the expression of specific genes are needed to stimulate this disease, reviewing recent findings on the existence of wheat susceptibility factors, the role of phytohormones on its development and the role of the effectors such as DON in the susceptibility.

## Genes and QTL Determining Resistance to Wheat Pathogens

Moderate to high levels of qualitative and quantitative resistance can be incorporated. Whereas qualitative resistance is almost complete, isolate specific and follows a gene for gene interaction (Brading et al., 2002), quantitative resistance is incomplete, polygenic, isolate-non-specific and more durable (Simón and Cordo, 1998).

For obtaining resistant cultivars, traditional breeding but also molecular tools to select plants according to the presence of specific genes and/or QTL are imperative. The QTL were originally mapped in biparental populations, restricted in allelic variations and with low genomic resolution. In recent years, some limitations of this traditional mapping were overcome by the genome-wide association studies (GWAS), which dissect the genetic basis of complex characters considering naturally occurring genetic variation providing higher mapping resolution (Korte and Farlow, 2013; Liu et al., 2016).

In this special issue, Babu et al. reviewed different strategies to control rusts, including the exploitation of complete and partial resistance, the pleiotropic effects of some resistance genes and the importance of molecular markers for the pyramidization of resistant genes in new varieties. Aspects of next-generation sequencing platforms and the development of the kompetitive allele-specific PCR (KASP) have been outlined. Additionally, the recent development of the sequence alignment of the genome and its importance to identify marker-trait associations, candidate genes and improved breeding values in studies of genomic selection were also summarized. The importance of advanced genotyping platforms to estimate genetic diversity and construction of the high-density genetic maps, isolation of genes and cloning were described.

Ghimire et al. reviewed that more than 500 QTL for resistance were reported for Fusarium head blight. Furthermore, 79 genes and more than 200 QTL were found for leaf rust and 82 genes and 140 QTL for stripe rust in the seedling and adult stages, respectively. Breeding efforts for incorporating resistance in the southeast of the USA mentioning the most used genes and QTL present in the current cultivars were reported. Carmack et al. found that optical sorter selection was useful to identify lines with resistant alleles for markers associated with some specific genes for Fusarium head blight, such as *Fhb1*, finding kernel damage and DON reductions after sorter selection.

Kumar et al. found 9, 18, and 26 QTL significantly associated with seedling resistance to two or more pathotypes of *P. striiformis, P. triticina*, and *P. graminis*, respectively, several of them for more than one rust type, in a panel of 483 spring wheats genotyped with a 35K SNP array in a GWAS. Furthermore, there were 16, 18, and 27 QTL associated with leaf rust, yellow rust and stem rust, respectively, in two out of four environments in the adult stage, some associated with more than one rust type. Candidate genes were also reported.

Huerta-Espino et al. detected that *Lr34, Lr45, Lr67*, and *Lr68*, conditioning resistance to leaf rust alone or in combinations, were present among 51 tall and semi-dwarf varieties released in Mexico before and after the Green Revolution, together with additional slow rusting and race-specific genes. Leaf necrosis associated with genes for slow rusting was evident in all cultivars and most of them showed the pseudo black chaff phenotype in the glumes and below the nodes (Borlaug et al., 1949) due to the presence of the gene *Str2*. Furthermore, associations of the slow rusting genes with durable resistance for leaf rust, yellow rust and stem rust, including the race Ug99, were found in cultivars released before the Green Revolution and their descendants.

Another important wheat disease affecting leaves, nodes and glumes is Septoria nodorum blotch caused by *Parastagonospora* (anamorph, *Stagonospora*; teleomoph, *Phaeosphaeria*) *nodorum* (Berk.) Quaedvlieg, Verkley, and Crous. Francki et al. detected significant genotype × environment interactions regardless of whether the same or different isolates were inoculated in the adult stage of 232 genotypes from diverse origins in Western Australia. However, several genotypes showed high levels of resistance against different isolates in multi-location trials. The GWAS also detected 20 QTL associated with the resistance on chromosomes 1A, 1B, 4B, 5A, 5B, 6A, 7A, 7B, and 7D, some of them novel and multiple against different isolates in some environments.

In the last years, new aggressive strains of *Puccinia striiformis* Westend caused epidemics in warmer regions across the world (Liu et al., 2017). Mu et al., using a GWAS, genotyped a panel of 857 USA genotypes with molecular markers for 18 resistance genes or QTL. They phenotyped the accessions in seedlings with six predominant or most virulent races of *Puccinia striiformis* and by natural infection in the adult stage, identifying 51 loci associated with resistance. Genes *Yr5* and *Yr15*, which are resistant to all races in the USA and *Yr 46*, resistant to many races, were not present.

Phuke et al. also conducted a GWAS using 11,401 SNP markers and 184 genotypes from CIMMYT and South Asia, phenotyped in seedlings with race 1 of *Pyrenophora tritici repentis* (Died.) Drechs, anamorph *Drechslera tritici-repentis* (Died), the causal agent of tan spot. They found QTL on chromosomes 1B, 2A, 2B, 3B, 4A, 5A, 5B, 6A, and 7D, several of them novel. Marker-trait associations on chromosomes 5AL and 5BL were consistent across environments, being the *Vrn–A1* locus identified on 5AL, whereas the marker on 5BL seems to correspond to gene *ts1*.

The pyramidization of major and minor genes could lead to a higher level and more durable resistance (Singh et al., 2014). But testing the effective function of each gene in this stack is essential as these genes can interfere with each other (Hurni et al., 2014) or reduce effectiveness in a certain genetic background (Hiebert et al., 2020). The *Avr* effectors can be used as probes to confirm the function of *R* genes in the absence of the pathogen (Vleeshouwers and Oliver, 2014). Several major genes for rust resistance and other diseases have been cloned. In this special issue, Zhang et al. indicated that 24 rust resistance genes have been cloned, most of them due to approaches summarized as target-sequence enrichment and sequencing pipelines. However, cloning of *Avr* effectors is scarce. Kangara et al. described a procedure to accelerate the *Avr* cloning, developing a mutant population of *Puccinia graminis tritici* spores and select for virulent mutants toward specific resistant genes.

## Genomic Selection and the Incorporation of Resistance in Wheat Cultivars

Genomic selection (GS) is a form of marker-assisted selection for predicting the genetic values of untested populations using genome-wide markers and phenotype information from observed populations for acceleration of genetic progress (Wang et al., 2018). In this special issue, Verges et al. confirmed that regional nurseries are useful as a source of lines to predict genomic estimated breeding values for Fusarium head blight for local breeding programmes. They also constructed an index to identify lines with low DON content and Fusarium head blight resistance.

Sharma et al. described the resurrection of a previously widely grown Indian cultivar, which succumbed due to leaf and stripe rust, summarizing how the incorporation of stripe rust resistance genes was carried out and the outcomes after the release of the new cultivar.

## Biological and Chemical Control of Wheat Diseases

Chakraborty et al. found that some secondary metabolites derived from a marine *Bacillus subtilis* strain inhibit mycelial growth, conidiogenesis, and conidial germination and produced morphological modifications in the germinated conidia of *Magnaporthe oryzae Triticum* pathotype and suppression of the wheat blast disease. Castro Tapia et al. reported that some strains of *Pseudomonas protegens* decreased by 16.8% the severity caused by the wheat crown and root rot pathogens *Gaeumannomyces graminis* var. *tritici* (Sacc.) Arx D. L. Olivier, *Rhizoctonia cerealis* van der Hoeven, and *Fusarium culmorum* (Wm.G.Sm) Sacc., whereas spikes m^−2^ and number of grains per spike increased.

In the context of integrated disease management, Carmona et al. reviewed the effectiveness of different fungicides to control *Puccinia striiformis* and a classification according to its mode of action, mobility, and impact on infection processes. Seed and foliar fungicide treatments and the optimal number and timing of application and its impact on the wheat yield were also summarized.

The papers brought together a wide range of aspects related to etiology, pathogen populations, resistance breeding and integrated management of fungal diseases of wheat to increase yield and wheat quality.

## Author Contributions

MS prepared the draft. AB and PS revised the draft and approved the final submission. All authors contributed to the article and approved the final version.

## Conflict of Interest

The authors declare that the research was conducted in the absence of any commercial or financial relationships that could be construed as a potential conflict of interest.

## Abbreviations

QTL: quantitative trait loci
GWAS: genome wide association studies.
